# Fecal carriage of *vanB* antibiotic resistance gene affects adipose tissue function under vancomycin use

**DOI:** 10.1080/19490976.2022.2083905

**Published:** 2022-06-13

**Authors:** Lars M. M. Vliex, Giang N. Le, Marina Fassarella, Dorien Reijnders, Gijs H. Goossens, Erwin G. Zoetendal, John Penders, Ellen E. Blaak

**Affiliations:** a Department of Human Biology, NUTRIM, School for Nutrition and Translational Research in Metabolism, Maastricht University Medical Center+, Maastricht, The Netherlands; b Department of Medical Microbiology, Maastricht University Medical Center+, Maastricht, The Netherlands; c Laboratory of Microbiology, Wageningen University & Research, Wageningen, The Netherlands

**Keywords:** Antibiotic resistance, metabolic health, obesity, gut microbiome, *vanB*, vancomycin, adipose tissue, insulin sensitivity

## Abstract

Detrimental consequences of antibiotic treatment may include long-lasting disruption of the gut microbiota. Previous studies found no negative effects of antibiotics on metabolic health, although individualized responses were observed. Here, we aimed to investigate the subject-specific response to vancomycin use in tissue-specific insulin sensitivity by stratifying individuals based on the presence of antibiotic resistance genes (ARGs) or opportunistic pathogens (OPs) in the baseline fecal microbiota. Quantitative Polymerase Chain Reaction (qPCR) was used to detect ARGs and OPs in DNA isolated from fecal samples of 56 males with overweight/obesity (Body Mass Index: 25–35 kg/m^2^) and impaired glucose metabolism (fasting plasma glucose ≥5.6 mmol/L and/or 2-hour glucose 7.8–11.1 mmol/L). A two-step hyperinsulinemic-euglycemic clamp was performed to determine tissue-specific insulin sensitivity. Abdominal subcutaneous adipose tissue (AT) gene expression was assessed using Affymetrix microarray. Gut microbial composition was determined using the Human Intestinal Tract Chip (HITChip) microarray. At baseline, the vancomycin resistance gene *vanB* was present in 60% of our population. In individuals that were *vanB*-negative at baseline, AT insulin sensitivity (insulin-mediated suppression of plasma free fatty acids) improved during vancomycin use, while it decreased among *vanB*-positive individuals (% change post versus baseline: 14.1 ± 5.6 vs. −6.7 ± 7.5% (*p* = .042)). The vancomycin-induced increase in AT insulin sensitivity was accompanied by downregulation of inflammatory pathways and enrichment of extracellular matrix remodeling pathways in AT. In the *vanB*-positive group, well-known *vanB*-carrying bacteria, *Enterococcus* and *Streptococcus*, expanded in the gut microbiome. In conclusion, microbiome composition and adipose tissue biology were differentially affected by vancomycin treatment based on fecal *vanB* carriage.

## Introduction

Antibiotics have been in use for almost a century to treat bacterial infections, which has resulted in a significant increase in life-expectancy. One downside of widespread antibiotic use, marked by overuse and misuse, is that it leads to the selection for and increased dissemination of bacteria resistant to antibiotics.^1^ Bacteria can become resistant to antibiotics via *de novo* mutations in their genome or via horizontal gene transfer of antibiotic resistance genes (ARG).^2^

Next to the selection pressure for antibiotic resistant bacteria, another downside of antibiotics is that they do not discern between pathogenic and health-beneficial bacteria. This is especially the case for the gut microbiota, the collection of all microbes in the gastrointestinal tract, as it can be significantly affected by (oral) antibiotics, leading to changes in microbial composition, which may even result in altered microbial functionality.^3,4^ Given the importance of the gut microbiome in host health, such antibiotic-induced perturbations may in turn negatively impact host gastro-intestinal and metabolic health.^5,6^ Crosstalk occurs between the gut microbiome and different organs in the body such as the liver, brain, adipose tissue (AT) and skeletal muscle. This communication is partly mediated via microbial metabolites such as short-chain fatty acids (SCFA) and bile acids (BA).^7,8^ Another important function of the gut microbiome is to strengthen host immunity. Microbial dysbiosis has been associated with impaired epithelial barrier function, resulting in increased translocation of bacteria and bacterial remnants into the bloodstream. This elicits a pro-inflammatory response as molecules such as lipopolysaccharide (LPS) interact with immune cells.^9^ On a similar note, opportunistic pathogens (OP) can take advantage of disturbances in gut microbial composition, leading to favorable conditions for these microbes to bloom. Especially in vulnerable hosts (e.g. hospitalized patients), this can lead to life-threatening infections.^10^ Disruption of a healthy gut microbiome may thus have consequences for the host’s immune system and metabolic health, as beneficial gut-derived metabolites may decrease while pro-inflammatory factors increase.

Vancomycin is a glycopeptide antibiotic that is used to treat infections with Gram-positive bacteria.^11^ It inhibits specific steps in the synthesis of the peptidoglycan layer, thereby leaving the bacteria susceptible to lysis.^12^ Previous work from our group showed that seven days of vancomycin treatment altered gut microbial composition in males with overweight or obesity and impaired glucose metabolism. This vancomycin-induced change persisted for up to eight weeks after treatment cessation.^13^ Conversely, a 7-day amoxicillin treatment did not affect gut microbial composition. The vancomycin-induced change in microbial composition was accompanied by alterations in fecal and plasma SCFA and BA levels but had no effect on tissue-specific insulin sensitivity, energy and substrate metabolism and systemic low-grade inflammation. Strikingly, vancomycin use altered abdominal subcutaneous adipose tissue (AT) gene expression toward a more oxidative phenotype, suggesting improvements in metabolic health.^13^ Moreover, the expression of genes related to inflammatory processes was decreased. Interestingly, in a study in adults with overweight or obesity without diabetes, upregulation of inflammatory pathways in subcutaneous AT was associated with peripheral insulin resistance.^14^ Thus, one may speculate that vancomycin use could have beneficial effects on AT function and insulin sensitivity through lowering AT inflammation.

In line with our previous findings showing no effect of vancomycin on host metabolism,^13^ a 14-day vancomycin treatment did not affect energy expenditure and substrate metabolism, glucose tolerance and insulin levels in adults with obesity and impaired glucose tolerance, although plasma SCFA and BA levels decreased.^15^ Furthermore, a 4-day treatment with an antibiotic cocktail including vancomycin did not affect postprandial glucose and insulin levels in healthy, normal weight men.^16^

Conversely, a study in men with obesity and metabolic syndrome showed decreased peripheral insulin sensitivity after seven days of vancomycin treatment, although effects were small and this study did not include a control group.^17^ Thus, the sparse evidence on the effect of vancomycin use on metabolic health is contradictory. It is, however, increasingly evident that the impact of antibiotic use on gut microbial composition and related effects on metabolic health, as well as on their recovery after antibiotic use, is subject-specific.^18^

The presence, abundance and diversity of ARGs in the microbiome is one factor that may explain subject-specific responses to antibiotic use. As bacteria carrying ARG are protected, they can expand under the usage of a specific antibiotic, while susceptible bacteria are inhibited and may perish. Individuals harboring these resistant bacteria might also react differently to antibiotic use. Moreover, ARGs encoding enzymes that inactivate antibiotics might not only protect the bacterial host but also benefit neighboring bacterial communities when secreted. On a similar note, certain OPs may take advantage of the opportunity created by antibiotic use, allowing these bacteria to expand as potential competitors are inhibited and nutrients become available. Thus, ARG and OP presence may modulate the response to antibiotic use in gut microbial composition and metabolic health. So far, only a few human intervention studies have investigated the effect of antibiotic use on metabolic health,^13,15–17^ and the potential modulating role of ARGs and OPs herein remains unexplored.

In this paper, we aimed to investigate the subject-specific response to antibiotic use by stratifying individuals based on the presence of specific ARGs or OPs in the baseline fecal microbiota. We hypothesized that ARGs or OPs would affect the response to antibiotic use in gut microbial composition and metabolic health, with a focus on adipose tissue metabolism and tissue-specific insulin sensitivity. It was expected that resistant bacteria would expand under antibiotic use, which in turn could lead to detrimental effects on metabolic health.

## Results

**Figure 1.**
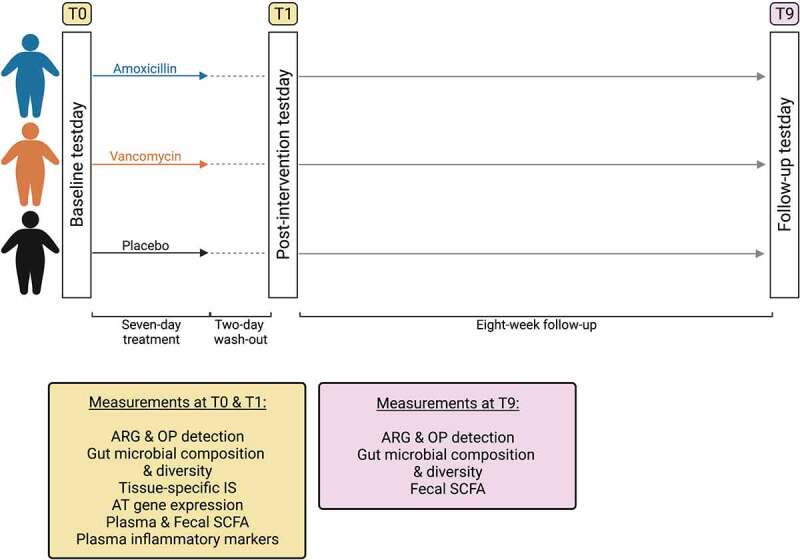
Study overview. Participants received a 7-day treatment with amoxicillin, vancomycin or placebo. Testdays took place at baseline (T0), after a 2-day wash-out after treatment cessation (T1) and after an 8-week follow-up (T9). Created with BioRender.com.

Participants received days of vancomycin, amoxicillin or placebo treatment (Figure 1). At baseline (T0), days (wash-out) after treatment cessation (T1) and subsequently at 8-week follow-up (T9), measurements were performed. At all testdays, gut microbial composition and diversity as well as the presence of ARG and OP in the gut microbiome were analyzed. Furthermore, levels of fecal SCFA were determined. At T0 and T1, tissue-specific insulin sensitivity was determined and levels of plasma inflammatory markers and plasma and fecal SCFA were analyzed. In addition to this, abdominal subcutaneous AT biopsies were taken to analyze AT gene expression.

### Presence of specific ARG and OP in the study population

We quantified common ARGs conferring resistance against vancomycin and β-lactam antibiotics, as well as the opportunistic pathogens *Clostridioides difficile* and *Escherichia coli*. The *vanB* vancomycin resistance gene and the *TEM* and *SHV* gene-families, conferring resistance against amoxicillin, were the most prevalent ARGs (Table 1). At baseline, *vanB* was present in 60% of our population (33/55). *CTX-M* genes, encoding extended-spectrum β-lactamases (ESBL), and CMY genes, encoding AmpC-type beta-lactamases, were only sparsely detected. *E. coli* was detected in almost every sample, while *C. difficile* was only detected sporadically. The four most prevalent ARGs (*vanB, TEM, SHV*) and OPs (*E. coli*) were further investigated to determine how their presence would change after antibiotic use and follow-up.

**Table 1.** Presence of antibiotic resistance genes and opportunistic pathogens in our study population at baseline, after seven days antibiotic treatment (week 1) and after eight-week follow-up (week 9)

*% (n)*	Amoxicillin			Vancomycin			Placebo		
	Baseline(n=18)	Week 1(n=17)	Week 9(n=17)	Baseline(n=19)	Week 1(n=19)	Week 9(n=17)	Baseline(n=18)	Week 1(n=19)	Week 9(n=17)
*vanA*	0	0	0	0	0	0	0	0	0
*vanB*	72 (13)	59 (10)	71 (12)	47 (9)	37 (7)	59 (10)	61 (11)	53 (10)	53 (9)
									
*CTX-M*	11 (2)	12 (2)	12 (2)	16 (3)	26 (5)	0	0	0	6 (1)
*TEM*	72 (13)	71 (12)	71 (12)	68 (13)	79 (15)	53 (9)	61 (11)	42 (8)	41 (7)
*SHV*	11 (2)	24 (4)	29 (5)	11 (2)	63 (12)	18 (3)	6 (1)	5 (1)	6 (1)
*CMY*	0	0	6 (1)	0	21 (4)	0	0	0	6 (1)
									
*C. difficile*	6 (1)	0	0	5 (1)	5 (1)	6 (1)	0	0	0
*E. coli*	94 (17)	88 (15)	94 (16)	100 (19)	100 (19)	94 (16)	94 (17)	84 (16)	94 (16)

CMY = CIT-type AmpCs

### vanB relative abundance increases after vancomycin use

**Figure 2.**
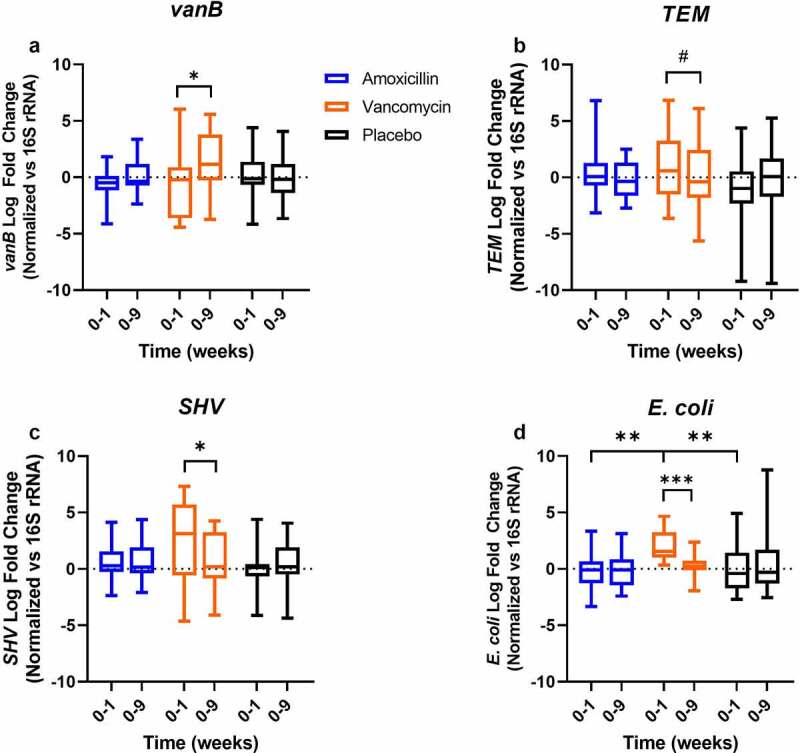
Log fold change of antibiotic resistance genes targets and *E. coli* after the 7-day amoxicillin, vancomycin or placebo treatment (week 0–1) and after 8-week follow-up (week 0–9). Data were normalized vs. 16S rRNA gene copy number (n = 17, 17, 19, 17, 18, 16 for Amoxicillin 0–1, 0–9, Vancomycin 0–1, 0–9, Placebo 0–1, 0–9, respectively). Differences within treatment groups were analyzed using paired t-test. Differences between treatment groups were analyzed using one-way ANOVA with post-hoc testing. *p < .05; #*p* < .1; **p < .001.

In the vancomycin-treated group, there was an increase in the relative abundance of *vanB* at follow-up (week 0–9), and this was significantly higher compared to after the 7-day vancomycin treatment (week 0–1) (fold change: 1.42 ± 0.62 vs. −0.44 ± 0.67; *p* = .029), indicating that *vanB* abundance mainly increased after cessation of antibiotic use (Figure 2a). For *TEM* and *SHV*, no significant differences were seen in the amoxicillin-treated group (Figure 2(b,c)). In the vancomycin-treated group however, relative abundance of *SHV* was significantly increased in week 0–1 compared to week 0–9 (2.61 ± 0.93 vs. 0.69 ± 0.62; *p* = .032), while relative abundance of *TEM* tended to be higher in week 0–1 (1.25 ± 0.75 vs. 0.15 ± 0.77; *p* = .062). In the vancomycin-treated group, the increase of *E. coli* was significantly higher in week 0–1 compared to week 0–9 (fold change: 2.20 ± 0.33 vs. 0.24 ± 0.26; *p* < .001) (Figure 2d). This temporary bloom of *E. coli* was only observed in the vancomycin-treated group, as confirmed by a significant effect of treatment on the increase in *E. coli* levels in week 0–1 (one-way ANOVA (F(2,51)=10.669; p<0.001))  . Post hoc comparisons showed a significant increase in the vancomycin-treated group compared to both amoxicillin and placebo (fold change: 2.20 ± 0.33 vs. −0.13 ± 0.43 or vs. 0.19 ± 0.55; both *p* < .001).

### AT insulin sensitivity improves in vanB-negative individuals during vancomycin use

In order to investigate the effect of ARGs on host metabolic health, the study population was stratified based on the presence of ARGs at baseline. As *vanB* was carried by 60% of the population at baseline, we focused our analyses on the presence (*vanB*1) or absence (*vanB*0) of *vanB*. Interestingly, the number of subjects carrying *vanB* decreased during vancomycin use (Table 1). At the 8-week follow-up, *vanB* was detected in 10 of the 17 individuals (59%), including two individuals with newly acquired resistance.

Baseline Body Mass Index (BMI) differed based on *vanB* presence in the vancomycin-treated group: the *vanB*-negative group had a higher BMI compared to *vanB*-positive (32.7 ± 0.66 vs. 30.1 ± 0.81 kg/m^2^; *p* = .020) (Table 2). Other characteristics did not differ between the subgroups.

**Table 2.** Baseline characteristics of the vancomycin-treated subgroup (n=19), split based on *vanB* presence at baseline

Baseline vancomycin-treated	*vanB*0			*vanB*1			*n*	
	Mean	SEM		Mean	SEM	*p*-value	*vanB*0	*vanB*1
Age (yrs)	62	1.9		59	2.4	0.315	10	9
Weight (kg)	99.6	2.31		95.2	3.15	0.270	10	9
BMI (kg/m^2^)	32.7	0.66		30.1	0.81	0.020*	10	9
Waist-to-hip ratio	1.08	0.18		1.06	0.02	0.408	10	8
Fasting glucose (mmol/L)	6.1	0.15		6.1	0.24	0.806	10	9
2hr glucose (mmol/L)	6.8	0.57		7.7	0.55	0.239	10	9
Insulin (mU/ml)	16.4	1.58		17.3	1.73	0.721	10	9
HOMA-IR	4.4	0.40		4.7	0.57	0.653	10	9
HbA1c (%)	5.5	0.09		5.7	0.15	0.298	10	9
Creatinine (µmol/L)	83.9	3.32		82.2	5.74	0.784	9	6
ALAT (U/L)	30.1	2.34		36.7	2.58	0.088	9	6
Hb (mmol/L)	9.7	0.24		9.5	0.16	0.507	6	8

Independent t test or Mann-Whitney U test were used to analyze difference between groups. *vanB*-negative at baseline (*vanB*0); *vanB*-positive at baseline (*vanB*1); Homeostatic model assessment for insulin resistance (HOMA-IR); Glycated hemoglobin (HbA1c); Alanine aminotransferase (ALAT); Hemoglobin (Hb).

**Figure 3.**
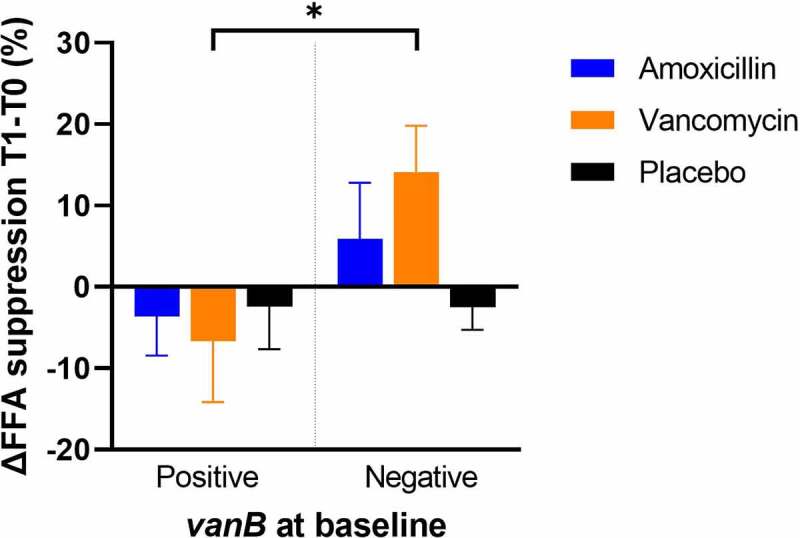
Changes in insulin-mediated suppression of FFA release (indicator of AT insulin sensitivity) after the 7-day amoxicillin, vancomycin or placebo treatment compared to baseline, with treatment groups split based on *vanB* presence at baseline (n = 12, 5, 9, 10, 11, 7 for amoxicillin *vanB*-positive, *vanB*-negative, vancomycin *vanB*-positive, *vanB*-negative, placebo *vanB*-positive, *vanB*-negative respectively). Differences within treatment groups between were analyzed using paired t-test. *p < .05.

To investigate the impact of ARG presence on metabolic health, changes in metabolic parameters during vancomycin use were compared between groups stratified based on the presence of *vanB* in baseline fecal samples. Remarkably, changes in AT insulin sensitivity (change in insulin-mediated suppression of free fatty acid (FFA) release) differed under vancomycin treatment (Figure 3). AT insulin sensitivity improved in the group that did not carry *vanB* at baseline (14.1 ± 5.57%), while it decreased in the *vanB*-positive group during vancomycin use (−6.7 ± 7.47%). When correcting for baseline BMI and AT insulin sensitivity, the difference in changes in AT insulin sensitivity remained significant (*p* = .042). No differences in the change in peripheral or hepatic insulin sensitivity were found during vancomycin use, although baseline peripheral insulin sensitivity (insulin-stimulated rate of glucose disappearance (RD)) was lower in the *vanB*-negative group (18.4 ± 2.30 vs. 26.3 ± 2.95 μmol⋅kg^−1^⋅min^−1^; *p* = .027) (Suppl. Figure 1 and Suppl. Table 1).

### Inflammatory pathways are downregulated in AT of vanB-negative individuals under vancomycin use

**Figure 4.**
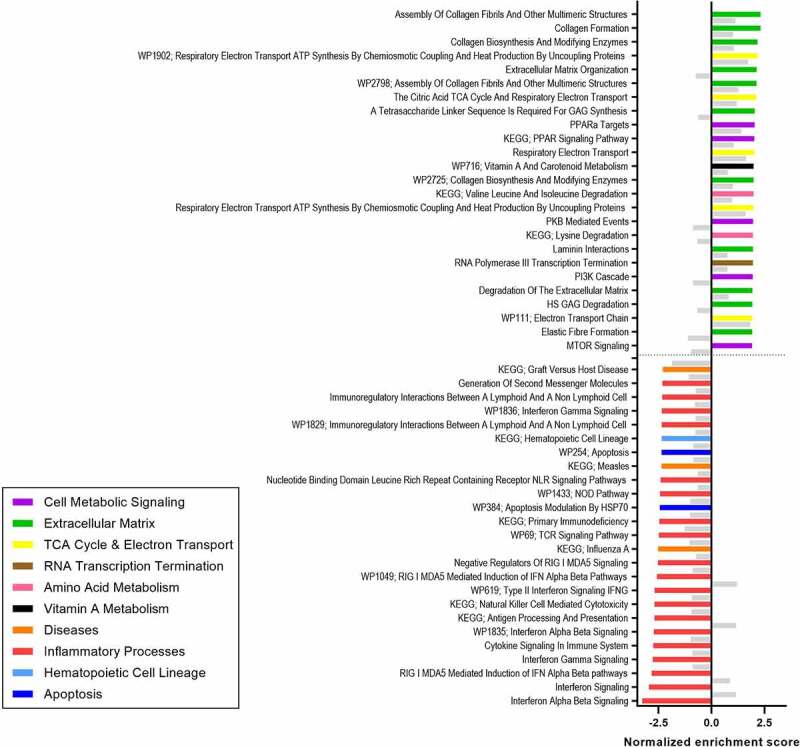
Top 25 upregulated and downregulated pathways in subcutaneous AT after vancomycin treatment compared to baseline in the *vanB*-negative at baseline (*vanB*0) group (FDRq < 0.1) (n = 5). Non-significant enrichment of these pathways in the *vanB*-positive group is given as a reference in gray (n = 6).

Next, the effect of vancomycin treatment on abdominal subcutaneous AT gene expression was analyzed using gene-set enrichment analysis. Changes in AT gene expression under vancomycin use differed based on carriage of *vanB* at baseline. In the *vanB*-negative group, 146 pathways were upregulated after vancomycin treatment, while 240 pathways were downregulated (FDRq < 0.1). The 146 upregulated pathways included multiple pathways related to extracellular matrix (ECM) remodeling and pathways related to mitochondrial function (Figure 4) (Suppl. Table 2 for a full list of significant pathways). Of the 240 downregulated pathways after vancomycin use, the majority were related to inflammatory processes (Suppl. Table 3 for a full list of significant pathways). In the *vanB*-positive group, no changes in gene expression were apparent after vancomycin treatment.

### Smaller changes in gut microbial diversity in vanB-negative individuals during vancomycin use

**Figure 5.**
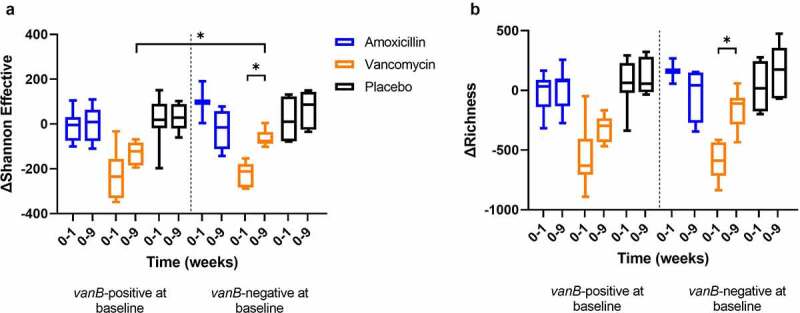
Changes in α-diversity indices (Shannon Effective (a) and richness (b)) between baseline and after the 7-day treatment (week 0–1), and between baseline and 8-week follow-up (week 0–9) with treatment groups split by *vanB* presence at baseline (*vanB*-positive: n = 10, 9, 7, 7, 9, 6; *vanB*-negative: n = 2, 4, 6, 6, 4, 6 for Amoxicillin 0–1, 0–9, Vancomycin 0–1, 0–9, Placebo 0–1, 0–9, respectively). Differences between *vanB*-positive and negative groups were analyzed using independent t-test. Differences within *vanB*-positive and negative groups were analyzed using paired t-test. *p < .05.

To investigate if these changes in AT function were linked to differences in gut microbial composition, changes in parameters of microbial diversity during vancomycin treatment were compared between *vanB0* and *vanB*1 groups. In the vancomycin-treated group, the decrease in Shannon Effective (Shannon Index converted to true diversity, a measure of α-diversity) from baseline to follow-up (week 0–9) was larger in *vanB*-positive individuals compared to *vanB-*negative (−113.5 ± 17.78 vs. −61.3 ± 18.20; *p* = .032), indicating a slower recovery after vancomycin use (Figure 5a).

**Figure 6.**
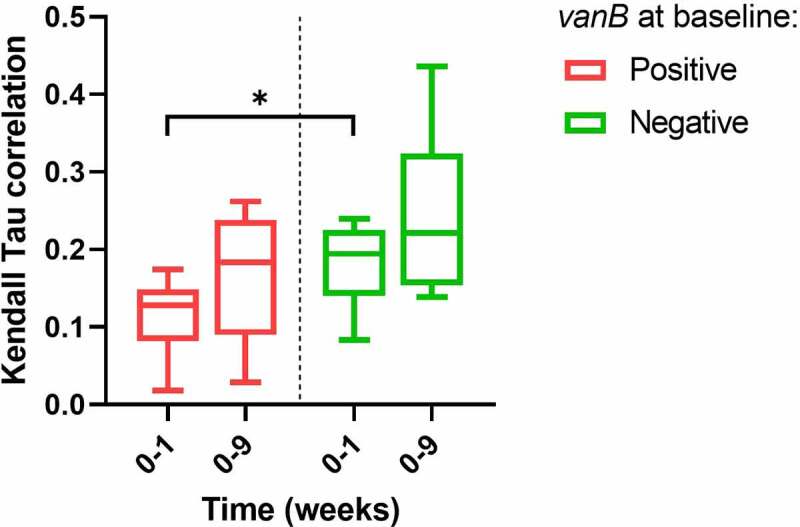
Kendall Tau correlation of sample similarity between baseline and after 7-day vancomycin treatment (week 0–1) and baseline and 8-week follow-up (week 0–9) as a measure of β-diversity, with treatment groups split by *vanB* presence at baseline (n = 7, 6 for *vanB*-positive and negative, respectively). Differences between *vanB*-positive and *vanB*-negative groups were analyzed using independent t-test. *p < .05.

Gut microbial community structure, here given as Kendall-Tau correlation as a measure of similarity, differed between groups: the within-subject similarity between samples collected at baseline and after 7-day vancomycin treatment (week 0–1) was lower in *vanB*-positive individuals compared to *vanB*-negative (0.11 ± 0.19 vs. 0.18 ± 0.02; *p* = .034), indicating increased dissimilarity after vancomycin use among individuals carrying *vanB* (Figure 6).

### Gut microbial composition changes under vancomycin use based on vanB presence

**Figure 7.**
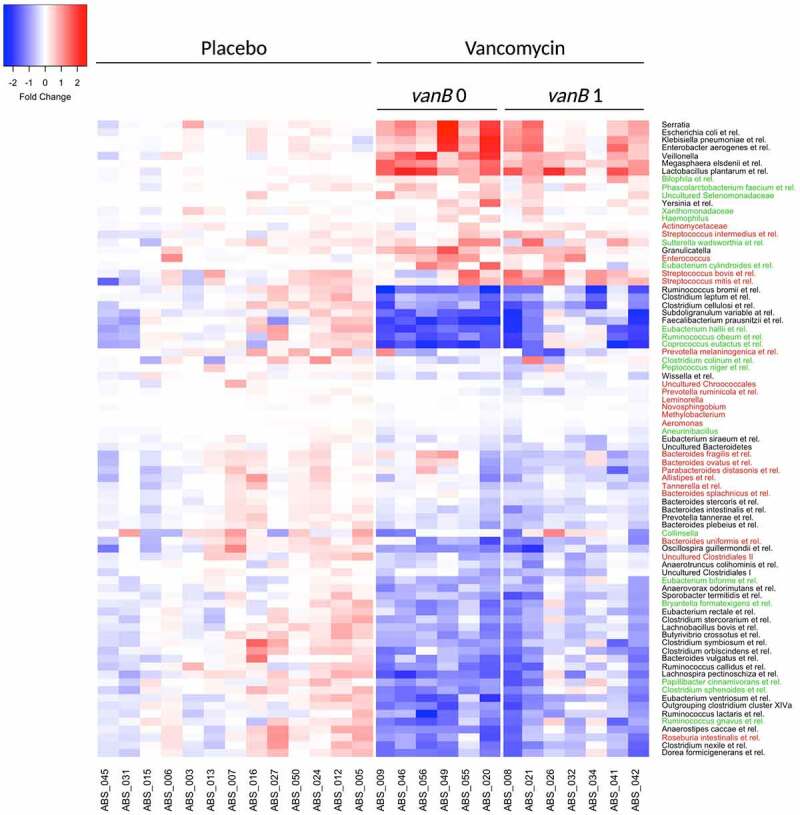
Heatmap of bacterial groups at genus-like level whose relative abundance was significantly different after 7-day vancomycin treatment compared to baseline, with treatment groups split by *vanB* presence at baseline (FDRq < 0.1). Wilcoxon SR test with Benjamini–Hochberg correction was used to analyze differences in bacterial groups over time. Color values show log_10_ fold changes compared to baseline. Color of genus-like groups indicates the subgroup in which the significant difference was found. Green: *vanB*0; red: *vanB*1; black: both groups.

Next, changes in gut microbial composition were assessed. In total, 81 genus-like bacterial groups were found to be significantly different after vancomycin treatment compared to baseline in either *vanB-*negative at baseline, *vanB-*positive, or in both groups (FDRq < 0.1) (Figure 7). Of the genus-like groups that were differentially affected in the subgroups, *Streptococcus intermedium et rel*., *S. mitis et rel*., *S. bovis et rel*. and *Enterococcus* all strongly increased in the *vanB*-positive group during vancomycin use. Species from the *Enterococcus* and *Streptococcus* genera are well-known *vanB*-carriers. Changes in total bacterial load did not differ between *vanB*-negative and *vanB*-positive during vancomycin use (Suppl. Figure 2).

To investigate whether *vanB* presence at baseline would impact recovery of affected taxa, changes in gut microbial composition during the 8-week follow-up period after vancomycin treatment were analyzed. There was no difference in changes of genus-like bacterial groups during the follow-up period between *vanB*-positive and *vanB*-negative, suggesting that the presence of *vanB* at baseline does not have an impact on the recovery of gut microbial composition after vancomycin use.

### Presence of vanB does not lead to changes in SCFA or inflammatory markers under vancomycin use

Next, possible links between changes in gut microbial activity and changes in metabolic health during vancomycin treatment were investigated by analyzing levels of microbial metabolites and inflammatory markers. Changes in fasting plasma inflammatory cytokines, as well as fasting plasma SCFA and fecal SCFA did not differ between *vanB*0 and *vanB*1 (Table 3). Furthermore, changes in levels of fecal SCFA did not differ between groups during the 8-week follow-up period (Suppl. Table 4).

**Table 3.** Concentrations of fasting plasma inflammatory cytokines, fecal SCFA and fasting plasma SCFA at baseline and after seven-day vancomycin treatment in groups split based on *vanB* presence at baseline

During vancomycin treatment(post-pre)		*vanB*0			*vanB*1			*n*	
		Mean	SEM		Mean	SEM	*p*-value	*vanB*0	*vanB*1
IL-6 (pg/ml)	Pre	1.2	0.14		0.8	0.17	0.406	10	9
	Post	1.1	0.14		1.0	0.16		10	9
IL-8 (pg/ml)	Pre	5.6	0.48		4.9	0.54	0.264	10	9
	Post	5.8	0.43		6.0	0.74		10	9
TNF-α (pg/ml)	Pre	3.0	0.24		2.6	0.12	0.597	10	9
	Post	3.0	0.13		2.7	0.15		10	9
									
Fecal Acetate (µmol/g)	Pre	52.8	4.13		46.2	4.64	0.905	10	8
	Post	26.2	3.83		18.5	4.46		10	8
Fecal Propionate (µmol/g)	Pre	14.6	1.60		14.4	2.63	0.211	10	8
	Post	11.6	1.59		7.8	1.80		10	8
Fecal Butyrate (µmol/g)	Pre	12.9	1.46		11.4	1.65	0.893	10	8
	Post	2.9	0.33		1.4	0.38		10	8
									
Plasma Acetate (µmol/L)	Pre	39.3	9.57		26.2	5.34	0.667	10	9
	Post	35.5	9.38		25.7	3.25		10	9
Plasma Propionate (µmol/L)	Pre	3.1	0.54		3.3	0.53	0.320	10	9
	Post	2.0	0.22		3.2	0.43		10	9
Plasma Butyrate (µmol/L)	Pre	1.1	0.24		0.9	0.28	0.445	10	9
	Post	0.6	0.12		0.6	0.12		10	9

Independent t test was used to analyze differences between groups. *p*-value is given for the comparison of change in parameter (post-pre vs. post-pre). *vanB*-negative at baseline (*vanB*0); *vanB*-present at baseline (*vanB*1); Interleukin 6 (IL-6); Interleukin 8 (IL-8); Tumor necrosis factor alpha (TNF-α).

### Changes in bacterial groups correlate with changes in AT insulin sensitivity in vanB-negative individuals under vancomycin use

Finally, possible links between changes in the microbiome and changes in AT function under vancomycin use were investigated. There was no correlation between change in gut microbial α- and β-diversity and change in AT insulin sensitivity (Suppl. Table 5). For the 19 genus-like bacterial groups that changed under vancomycin treatment in the *vanB*0 group (Figure 7), two were correlated with change in AT insulin sensitivity. Change in *Eubacterium* biforme *et rel*. was positively correlated with the change in AT insulin sensitivity (Rho = 0.943, *p* = .005), while the change in *Eubacterium hallii et rel*. was negatively correlated with change in AT insulin sensitivity (Rho = −0.886, *p* = .019). Overall, both genus-like groups *Eu. biforme* and *Eu. hallii* decreased in *vanB0* during vancomycin use. No correlations were found in the *vanB*1 group.

## Discussion

The aim of this study was to investigate the subject-specific response to antibiotic use by stratifying individuals based on the presence of ARGs or OPs at baseline. We found that the presence of *vanB* in the baseline microbiota impacted the response to vancomycin treatment in gut microbial composition, AT insulin sensitivity and AT gene expression in men with overweight or obesity and impaired glucose metabolism.

The presence of specific ARGs and OPs in the study population was analyzed. *E. coli* was present in almost all samples, as expected, as it is a common inhabitant of the human gut. Upon vancomycin use, there was a temporary bloom of *E. coli* that returned to baseline levels weeks after cessation of treatment. Vancomycin is not effective against Gram-negative *E. coli*, as the outer membrane of these bacteria prevents the antibiotic from reaching the peptidoglycan layer.^12^ Literature has shown expansion of the *Escherichia* genus after oral vancomycin use, as these bacteria can take advantage of a decrease in other species to expand.^19,20^ While *E. coli* should in theory be susceptible to amoxicillin, this was not observed here, a finding that may be explained by the fact that amoxicillin is well absorbed in the small intestine and may not reach the colon in sufficient amounts to impact the microbiome.^21^ This would also explain why there was no increase in the relative abundance of the *TEM* and *SHV* gene-families under amoxicillin use, contrary to expectations. Relative abundance of *TEM* and *SHV* gene families did increase after vancomycin use, which could be linked to the expansion of *E. coli* in our population, as these bacteria often carry members of the *TEM* or *SHV* gene families.^22^

*vanB* was present in 60% of individuals at baseline. The *vanB* gene confers resistance to vancomycin by modifying peptidoglycan precursors, the target site of vancomycin, thereby drastically lowering its ability to bind.^23^ In the vancomycin-treated group, relative abundance of *vanB* was significantly higher between week 0–9 compared to week 0–1, indicating expansion of *vanB*-carrying bacteria in the period after vancomycin use. Interestingly, this bloom did not occur directly during the week of vancomycin use. It could be that susceptible bacteria, capable of producing nutrients for *vanB*-carriers, were disrupted in this period, thereby inhibiting growth of the resistant bacteria. This way, cross-feeding within bacterial networks could play an important role in the response to antibiotic use.^24^ However, whether this did occur in our samples remains unclear.

During vancomycin treatment, AT insulin sensitivity increased in the group that did not carry *vanB* at baseline, while it decreased in the group carrying *vanB*, also after correction for differences in baseline BMI and AT insulin sensitivity. This indicates that vancomycin treatment may have a positive effect on AT metabolism and insulin sensitivity in specific subgroups of individuals. Concomitantly, changes in abdominal subcutaneous AT gene expression were found during vancomycin use in the *vanB*-negative group, as pathways related to inflammatory processes were downregulated after vancomycin treatment compared to baseline, while pathways involved in ECM remodeling and in mitochondrial function were upregulated. These findings imply positive effects of vancomycin on AT function in the absence of *vanB*. Both low-grade inflammation and limited ECM remodeling are associated with AT dysfunction in obesity: white adipocytes aim to expand in periods of energy excess, but their expandability is limited by low ECM remodeling.^25^ This may lead to adipocyte cell death and thus trigger local inflammation. If left unresolved, this may in turn lead to the development of insulin resistance.^26^ Thus, upregulation of ECM remodeling together with downregulation of AT inflammatory pathways may indicate a better expandability of adipocytes, preventing a stress response and local inflammation, which may in turn relate to higher AT insulin sensitivity in the *vanB*-negative group. These seemingly beneficial changes are further supported by the upregulation of pathways related to mitochondrial function in these individuals. Mitochondrial function is an important factor in healthy AT as it improves energy metabolism, and it has been associated with improved insulin sensitivity.^27^

In summary, we show that the absence of *vanB* in the gut microbiome may lead to improvements in AT function under vancomycin use, while the presence of *vanB* has detrimental consequences. Interestingly, these findings seem to be tissue-specific, as no changes in either peripheral or hepatic insulin sensitivity were found.

To elucidate the potential mechanisms underlying these changes in AT function, gut microbial composition and microbial diversity parameters were analyzed. There was no difference in α-diversity parameters during vancomycin use between subgroups, although an improved recovery in the Shannon Effective Index was observed at 8-week follow-up among subjects that did not carry *vanB*. In addition to improved recovery of α-diversity, a higher similarity between samples at baseline and after vancomycin use was found in the group that did not carry *vanB* at baseline, implicating reduced disruption by vancomycin use in the *vanB*-negative group. Changes in gut microbial composition differed between the groups during vancomycin use, as *Enterococcus* and *Streptococcus* expanded, but only in the group carrying *vanB* at baseline. This change was expected, as these Gram-positive bacteria have been shown to carry *vanB*.^28,29^ Of these, especially *Enterococcus* strains are often found to be the underlying cause of resistant infections in hospitals.^30^ Of note, changes in total bacterial numbers did not differ between *vanB*0 and *vanB*1 during vancomycin use.

One may speculate that in the *vanB*-negative group the lesser vancomycin-induced disruption led to the observed metabolically beneficial changes in AT gene expression profile and insulin sensitivity. This lower disruption may have led to altered production of microbial metabolites, such as SCFA and BA, which could positively impact metabolic health. However, in this study, no differences were found in either plasma SCFA, fecal SCFA or plasma inflammatory marker levels during vancomycin use between the *vanB*-negative and *vanB*-positive groups. It thus seems that these metabolites and cytokines are not the link between the changes in gut microbial composition and changes in AT insulin sensitivity observed here. Of note, the SCFA measured are just a small subset of the complex array of metabolites produced by the microbiome.^31,32^ To further elucidate the link between vancomycin-induced changes in gut microbial composition and tissue-specific functional changes in AT, extensive integrative metagenomic-metabolomic analyses of both fecal and/or plasma samples could prove useful.

Ultimately, there was no correlation between changes in gut microbial diversity parameters and AT insulin sensitivity under vancomycin use. However, in the *vanB*-negative group, change in *Eu. biforme et rel*. was positively correlated with change in AT insulin sensitivity, while *Eu. hallii et rel*. was negatively correlated. Both genus-like groups decreased under vancomycin use in *vanB*0. Drawing decisive conclusions based on these findings is difficult, however, as sample size is low and the *Eubacterium* genus is phylogenetically and phenotypically diverse. Our findings do indicate that specific changes in gut microbial composition may underlie the improvement in AT insulin sensitivity during vancomycin use. Further in-depth analysis of gut microbial composition is warranted to elucidate the changes occurring under vancomycin use. Combining this with resistome analysis to discover other potentially relevant ARGs should prove helpful in determining the role-resistant bacteria play in response to vancomycin use.

In conclusion, the present study demonstrates that microbial composition as well as adipose tissue metabolism and function were differentially affected by vancomycin treatment in men with overweight or obesity and impaired glucose metabolism carrying the *vanB* resistance gene in their gut microbiome compared to *vanB*-negative individuals. AT function improved in the *vanB*-negative group, along with tissue-specific insulin sensitivity. This was further substantiated by an improved gene expression profile, as pathways of ECM remodeling and mitochondrial function were enriched, while inflammatory processes were downregulated. Interestingly, the improvements seemed to be AT-specific. Furthermore, we showed a subject-specific response to vancomycin use caused by *vanB* presence. Whether similar effects occur with administration of other antibiotics should be analyzed further. Nevertheless, this study shows the potential of ARGs to modulate the response to antibiotic use for both gut microbial composition and metabolic health.

The subject-specific response to antibiotic use may prove important for future research, and identification of relevant ARGs could be an important first step to elucidate subject-specific response to antibiotic use that may be able to explain changes in metabolic health, specifically AT. This could be especially relevant in metabolically compromised populations. However, more research is needed to fully understand the impact of ARGs on metabolic health under antibiotic use and to unravel potential mechanistic links connecting these.

## Materials and methods

### Study design and population

The study population consisted of 56 adult males (age: 35–70 years) with overweight/obesity (BMI: 25–35 kg/m^2^) and impaired glucose metabolism (fasting glucose >5.6 mmol/l, and/or 2 hour glucose 7.8–11.0 mmol/l) and HOMA-IR >2.2, as described previously (https://ClinicalTrials.gov, NCT02241421).^13^ All participants gave written informed consent for participation in the study. The study was reviewed and approved by the local Medical Ethical Committee of Maastricht University Medical Center+. All procedures were performed according to the declaration of Helsinki (October 2008).

Participants were randomized to oral intake of amoxicillin, vancomycin or placebo (microcrystalline cellulose) for seven consecutive days (1500 mg/day). Block randomization with stratification for BMI, age and 2-hour plasma glucose values were used to ensure equal groups. Testdays took place at baseline, days (wash-out) after 7-day antibiotic or placebo treatment, and subsequently at 8-week follow-up (Figure 1).

### Detection of ARG and OP

Fecal samples were collected at the measurement days, and DNA was isolated as described previously.^33^ TaqMan quantitative PCR (qPCR) assays were used to determine the presence of specific ARGs in bacterial DNA. Quantification of the vancomycin resistance conferring gene *vanA* was done using a 5’-GCCGGAAAAAGGCTCTGAA-3’ forward and 5’-TCCTCGCTCCTCTGCTGAA-3’ reverse primer, generating a product of 67 bp, together with a 5’-ACGCAGTTATAACCGTTCCCGCAGACC-3’ probe with a 6-FAM-reporter and blackhole quencher (BHQ-1).^34^ For quantification of *vanB*, the 5’-CGCAGCTTGCATGGACAA-3’ and 5’-GGCGATGCCCGCATT-3’ forward and reverse primers were used, generating a 58 bp product, with a 5’-TCACTGGCCTACATTC-3’ probe with VIC-reporter and MGB-NFQ non-fluorescent quencher.^34^ Amplifications were performed using a Quantstudio 5 real-time PCR system (ThermoFisher Scientific) in 25 µl reactions containing 12,5 µl 2x Absolute qPCR ROX mix (ThermoFisher Scientific), 20 pM of each primer (*vanA* and *vanB*), 5 pM of probe (*vanA* and *vanB*) and 5 µl target DNA. Thermal cycling consisted of 15 minutes at 95°C, followed by 42 cycles of 15 seconds at 95°C and 30 seconds at 60°C. In order to assess the efficiency of the assays and to quantify the number of target copies in biological samples, standard curves were created using a control plasmid constructed by cloning the corresponding PCR amplicon into a p1190 (*vanA*) or p1200 (*vanB*) vector. Standard curves were made using triplicate measurements of serial dilutions of the control plasmid.

The presence of *Escherichia coli* and *Clostridioides difficile* was analyzed by qPCR, using previously validated assays.^35,36^ For the detection of the β-lactamase families *CTX-M, TEM, SHV* and CIT-type AmpCs, a multiplex qPCR setup was used based on work from Roschanski et al.^37^ These analyses are described in detail in the supplemental methods.

### Analysis of metabolic parameters and gut microbial composition

The following measurements were all performed in the original study.^13^ Tissue-specific insulin sensitivity was measured via two-step hyperinsulinemic-euglycemic clamp with [6,6–^2^H_2_]-glucose tracer analysis.^13,38^ Blood samples were taken from a superficial dorsal hand vein throughout the test. Tracer infusion was started at 0.04 mg/kg/min after bolus-injection (2.4 mg/kg) and was continued throughout the measurement. Variable co-infusion of 17.5% glucose solution, enriched with 1.1% tracer, was used to maintain plasma glucose concentrations at 5.0 mmol/L during the 0 mU, 10 mU/m^2^/min and 40 mU/m^2^/min insulin infusion steps (all 2 hours each). During the 10 mU insulin-infusion step, insulin-mediated suppression of FFA release and insulin-mediated suppression of endogenous glucose production (EGP) as measures of AT and hepatic insulin sensitivity were determined compared to baseline. Rate of glucose disappearance (RD) as a measure of peripheral insulin sensitivity was determined during the 40 mU insulin-infusion step. Blood samples were taken in the last 30 minutes of each step, in order to calculate steady-state kinetics:^38^

% FFA suppression = 100 – (([FFA at 10 mU] × 100)/[FFA 0 mU))

% EGP suppression = 100 – ((EGP at 10 mU × 100)/EGP at 0 mU)

(EGP = Rate of glucose appearance (RA) – Glucose infusion rate (GIR))

RD = RA + [Glucose tracer infusion]

Plasma glucose and FFA levels were determined using Cobas FARA auto-analyzer (Roche, Switzerland). Plasma insulin was determined using double antibody radioimmunoassay (Millipore). Additional fasting blood samples were collected, and plasma Interleukins (IL-6 and IL-8) and Tumor-Necrosis Factor Alpha (TNF-α) were measured using a multiplex enzyme-linked immunosorbent assay (ELISA) (Meso Scale Diagnostics). Plasma SCFAs were determined via liquid chromatography-tandem mass spectrometry.^13^

Abdominal subcutaneous AT biopsies were taken under local anesthesia in fasting conditions. RNA was isolated using Trizol chloroform extraction and was used for microarray analysis.^13^ Here, 100 ng RNA, labeled with Whole-Transcript Sense Target Assay, was hybridized to human whole-genome Affymetrix Gene 1.1 ST arrays, targeting 19793 unique genes (Affymetrix).

Gut microbial composition was determined using the Human Intestinal Tract Chip (HITChip) phylogenetic microarray.^39^ Fecal SCFA levels were determined using gas chromatography-mass spectrometry.^13^

### Statistical analyses

Data are expressed as mean ± SEM where possible, with a significance level of *p* < .05. Analyses were performed in IBM SPSS Statistics 27 and R version 4.0.5. Normality of data was assessed using Kolmogorov-Smirnov and visual inspection where necessary. Non-parametric equivalents of statistical tests were used in case normality was violated.

For the determination of ARG presence, a cutoff of 10 gene copies was used. Differences in ARG and OP fold change between treatments were analyzed using repeated measures ANOVA with multiple comparison. Differences within treatment were analyzed using paired t-test. Differences in parameters of metabolic health between groups were analyzed using independent t-test. Tissue-specific insulin sensitivity data were log10-transformed prior to analysis. In case of significant differences at baseline, ANCOVA was used to correct for covariates.

For the analysis of AT gene expression, gene set enrichment analysis (GSEA v4.1.0, UC San Diego) was performed using pre-ranked t values of genes from moderated (Bayesian smoothing) t-test (IBMT), computed with the Limma R package. Pathways were deemed significantly enriched when FDRq < 0.1.

Differences in gut microbial α- and β-diversity parameters between the groups were analyzed using independent t-test. Differences within groups were analyzed using paired t-test.

For the analyses of gut microbial composition using HITChip data, log10-transformed signals were used. Wilcoxon SR test with Benjamini–Hochberg correction (FDRq < 0.1) was used to compare changes in genus-like bacterial groups during vancomycin treatment and during 8-week follow-up within groups.

## Supplementary Material

Supplemental Material

## Data Availability

Data can be made available by the corresponding author upon reasonable request. Microarray data of the original study has been submitted to GEO: GSE76003. The script used here for HITChip analysis and visualization has been uploaded to GitHub: https://github.com/MUMC-MEDMIC/Lars_vancomycin

## Abbreviations

ARG: Antibiotic Resistance Gene; AT: Adipose Tissue; BA: Bile Acid; BMI: Body Mass Index; ECM: Extracellular Matrix; EGP: Endogenous Glucose Production; ELISA: Enzyme-linked Immunosorbent Assay; ESBL: Extended-spectrum Beta-lactamase; FFA: Free Fatty Acid; HITChip: Human Intestinal Tract Chip; IL-6: Interleukin-6; IL-8: Interleukin-8; LPS: Lipopolysaccharide; OP: Opportunistic Pathogen; qPCR: quantitative Polymerase Chain Reaction; RA: Rate of glucose appearance; Rate of glucose disappearance; SCFA: Short-Chain Fatty Acid; TNF-α: Tumor Necrosis Factor-alpha.
