# Supplementary material for: Fecal carriage of vanB antibiotic resistance gene affects adipose tissue function under vancomycin use
Source: Gut Microbes. 2022 Jun 13;14(1):2083905. doi: 10.1080/19490976.2022.2083905 (PMC9196849; doi:10.1080/19490976.2022.2083905)
Supplement: Supplemental Material [file KGMI_A_2083905_SM0269.zip › Supplementary Methods.docx]

**Supplemental methods**

***Detection of ARG and OP***

All TaqMan qPCR assays were performed using a Quantstudio 5 real-time PCR system (Thermofisher Scientific). The assays for *Clostridioides difficile* and *Escherichia coli* were performed separately. A multiplex qPCR assay was used for detection of *TEM, SHV, CTX-M* and CIT-type *AmpC*s.

***Clostridioides difficile***

Forward: 5’-TTGAGCGATTTACTTCGGTAAAGA-3’

Reverse: 5’-TGTACTGGCTCACCTTTGATATTCA-3’

Product: 151 bp

Probe: 5’-CCACGCGTTACTCACCCGTCCG-3’(rev) with FAM-reporter and TAMRA-quencher.

25 µl reaction

2x TaqPath qPCR MasterMix 12.5 µl

10 µM of Fw and Rev primer

10 µM Probe

5 µl Target DNA

Thermal cycling:

2 minutes at 95°C, 42 cycles of 15 seconds at 95°C and 60 seconds at 60°C.

***Escherichia coli***

Forward: 5’-CATGCCGCGTGTATGAAGAA-3’

Reverse: 5’-CGGGTAACGTCAATGAGCAAA-3’

Product: 96bp

Probe: 5’-TATTAACTTTACTCCCTTCCTCCCCGCTGAA-3’ with FAM-reporter and blackhole quencher (BHQ-1)

25 µl reaction:

2x TaqPath qPCR MasterMix 12.5 µl

10 µM of Fw and Rev primer

10 µM Probe

5 µl Target DNA

2 minutes at 95°C, 42 cycles of 15 seconds at 95°C and 60 seconds at 60°C.

***Multiplex qPCR assay***

***TEM***
Forward: 5’-GCATCTTACGGATGGCATGA-3’

Reverse: 5’-GTCCTCCGATCGTTGTCAGAA-3’

Product: 101bp

Probe: 5’-CAGTGCTGCCATAACCATGAGTGA-3’ with FAM-reporter QSY-quencher.

***SHV***
Forward: 5’-TCCCATGATGAGCACCTTTAAA-3’

Reverse: 5’-TCCTGCTGGCGATAGTGGAT-3’

Product: 105bp

Probe: 5’-TGCCGGTGACGAACAGCTGGAG-3’ with VIC-reporter and QSY-quencher.

***CTX-Ma***

Forward: 5’-CGGGCRATGGCGCARAC-3’

Reverse: 5’-TGCRCCGGTSGTATTGCC-3’

Product: 105bp

Probe: 5’-CCARCGGGCGCAGYTGGTGAC-3’ with ABY-reporter and QSY-quencher.

***CTX-Mb***

Forward: 5’-ACCGAGCCSACGCTCAA-3’

Reverse: 5’-CCGCTGCCGGTTTTATC-3’

Product: 221bp

Probe: 5’-CCCGCGYGATACCACCACGC-3’ with ABY-reporter and QSY-quencher.

***CMY***

Forward: 5’-GGCAAACAGTGGCAGGGTAT-3’

Reverse: 5’-AATGCGGCTTTATCCCTAACG-3’

Product: 101bp

Probe: 5’-CCTACCGCTGCAGATCCCCGATG-3’ with JUN-reporter and QSY-quencer.

For all targets the 25 µl reaction was done:

2x TaqPath qPCR MasterMix 12.5 µl

10 µM of Fw and Rev primer

10 µM Probe

5 µl Target DNA

2 minutes at 95°C, 42 cycles of 15 seconds at 90°C, 15 seconds at 50°C and 30 seconds at 60°C.
