# Supplementary figures and images for: Fecal carriage of vanB antibiotic resistance gene affects adipose tissue function under vancomycin use
Source: Gut Microbes. 2022 Jun 13;14(1):2083905. doi: 10.1080/19490976.2022.2083905 (PMC9196849; doi:10.1080/19490976.2022.2083905)

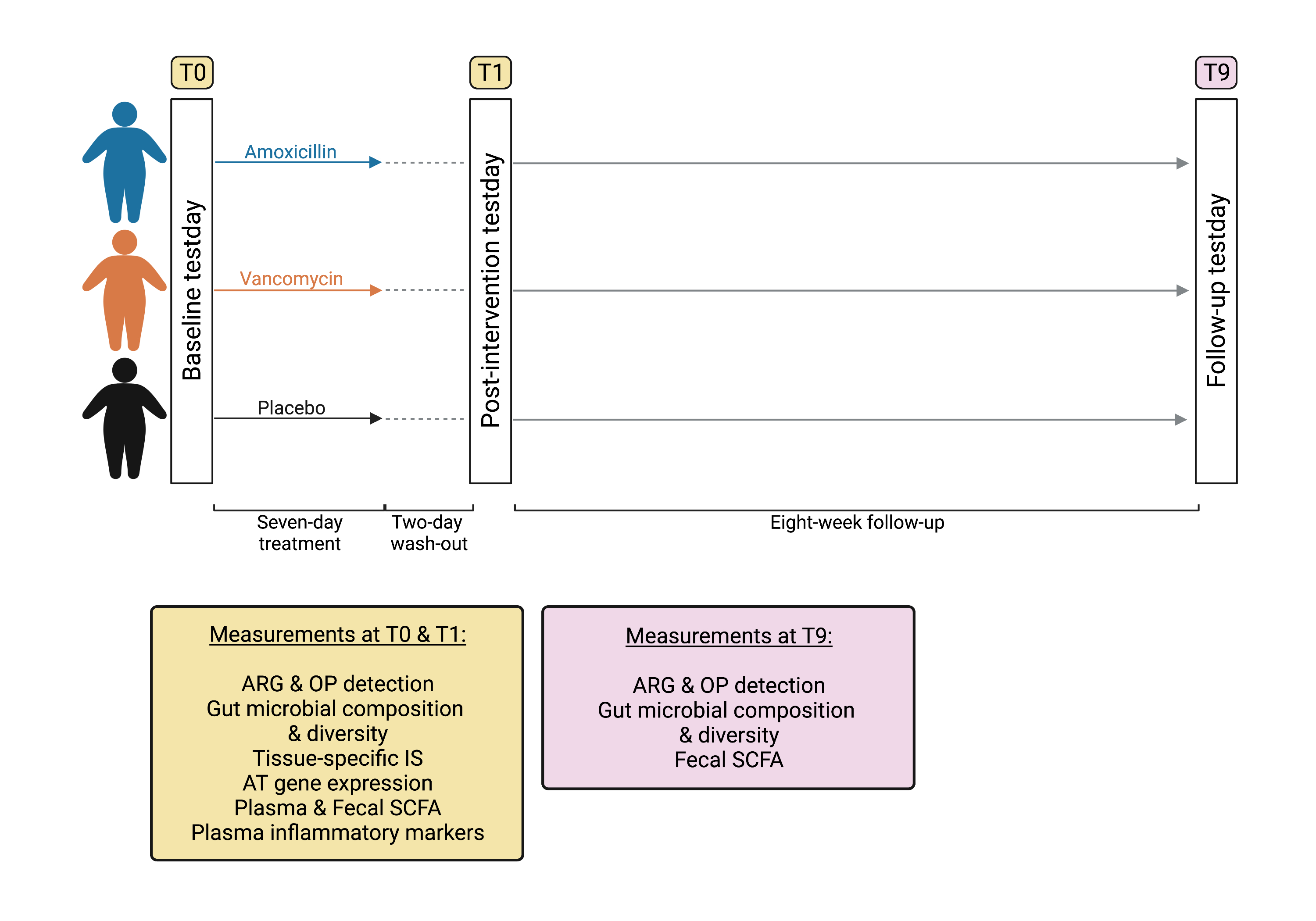

Supplement: Supplemental Material [file KGMI_A_2083905_SM0269.zip › Suppl_Figure 1.jpg]

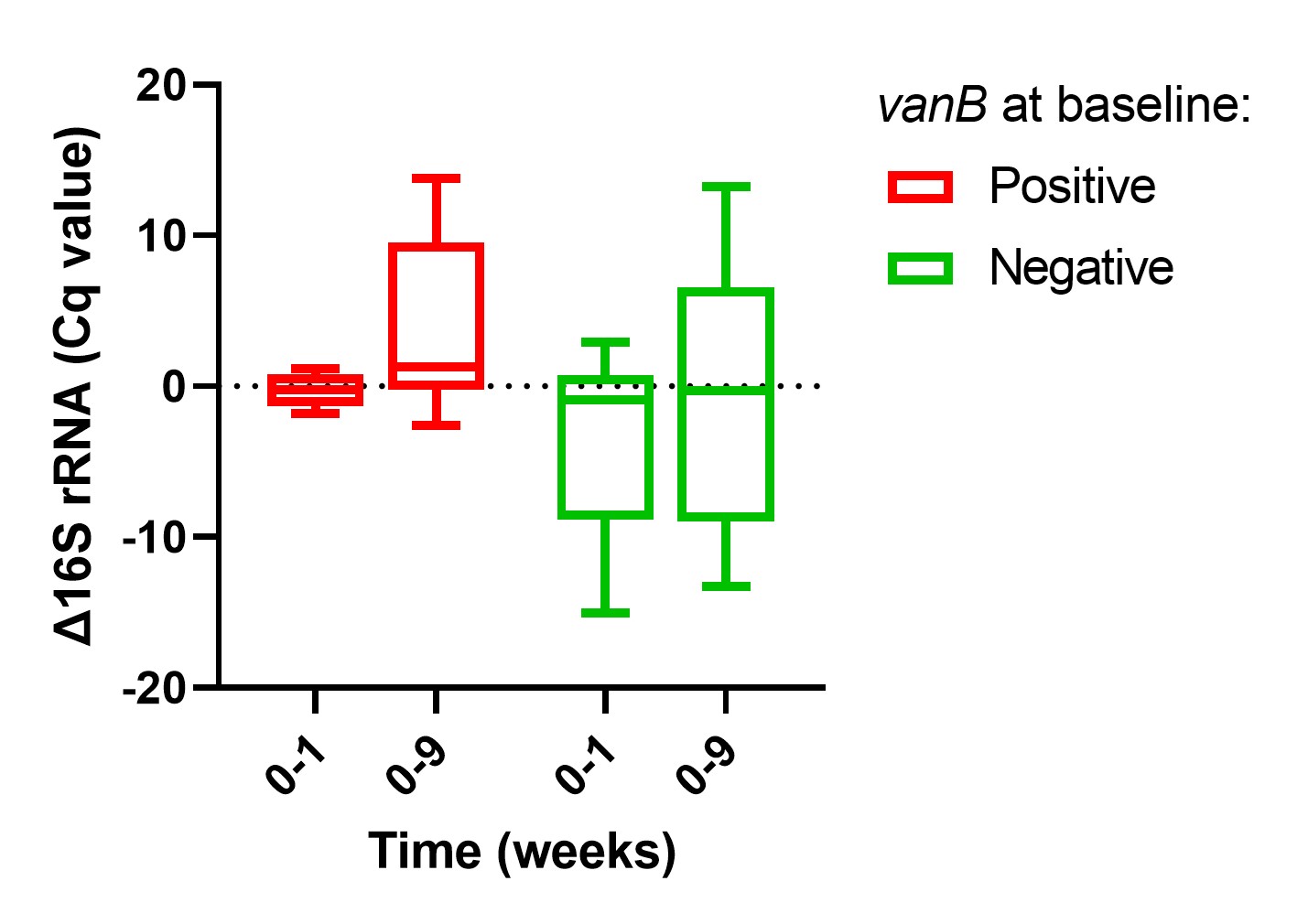

Supplement: Supplemental Material [file KGMI_A_2083905_SM0269.zip › Suppl_Figure 2.jpg]
